# Developing cell biology

**DOI:** 10.7554/eLife.00571

**Published:** 2013-02-19

**Authors:** Daniel Needleman

**Affiliations:** 1 **Daniel Needleman** is at the School of Engineering and Applied Sciences, the Department of Molecular and Cellular Biology, and the Center for Systems Biology, at Harvard University, United Statesdan.needleman@gmail.com

**Keywords:** mitotic spindle, embryogenesis, microtubule, kif2a, intracellular scaling, mitosis, Xenopus

## Abstract

Experiments in *Xenopus* embryo extracts reveal that changes in cellular biochemistry cause mitotic spindles to decrease in size over the course of early development.

**Related research article** Wilbur JD, Heald R. 2013. Mitotic spindle scaling during *Xenopus* development by kif2a and importin α. *eLife* **2**:e00290. doi: 10.7554/eLife.00290

**Image** The mitotic spindle (red) segregates chromosomes (blue) during cell division

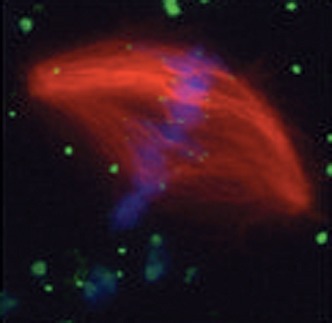

One of the many fibs our teachers told us for our own good is that animals are all the same at the cellular and molecular level despite their apparent outward differences. Thinking in that mindset, it's okay to study how ‘the cell' works without worrying too much about which particular cell one is considering. In addition to being helpful when preparing for exams in introductory biology courses, this fiction has practical benefits for scientists. Focusing on the features that cells have in common has enabled researchers to make great advances by studying the organisms most amenable to their question and method of choice, and to synthesize information to obtain coherent pictures of biological processes. In reality, there is variation throughout all of biology, including at the cellular and molecular levels, and even the most basic cellular processes, such as the mechanics of cell division, can differ dramatically between animal cells. There can even be large variations between different cells of the same organism: it has been known for more than a hundred years that spindles, the structures that segregate chromosomes during cell division, show great variations in size and shape over the course of development (Wilson, 1897). Very little is understood about the origins of this variation—which might have important implications for development, evolution and human health. Now, writing in *eLife*, Jeremy Wilbur and Rebecca Heald of the University of California at Berkeley offer important insights into the mechanisms that underlie these changes in spindle morphology (Wilbur and Heald, 2013).

Embryos undergo multiple rounds of rapid division during early development in many animals, and as the cells become progressively smaller, so too do the spindles that are responsible for their divisions (Figure 1). Recent studies of these early divisions in *Xenopus laevis* (Wuhr et al., 2008), *C. elegans* (Hara and Kimura, 2009; Greenan et al., 2010) and mouse (Courtois et al., 2012) have produced many interesting results, but the underlying causes of these changes in spindle size remain unclear. Since both cell size and spindle size decrease, it is tempting to think that there is some causative relationship between the two phenomena: that the confines of a smaller cell make spindles smaller, perhaps due to mechanics (Hara and Kimura, 2009) or the depletion of a limiting pool of cytoplasmic components (Goehring and Hyman, 2012). However, this is not the only possibility. It could be that changes in cellular biochemistry during early development give rise to smaller spindles through processes that are not connected to cell size. Understanding which of these scenarios is correct has been challenging.

**Figure 1.**
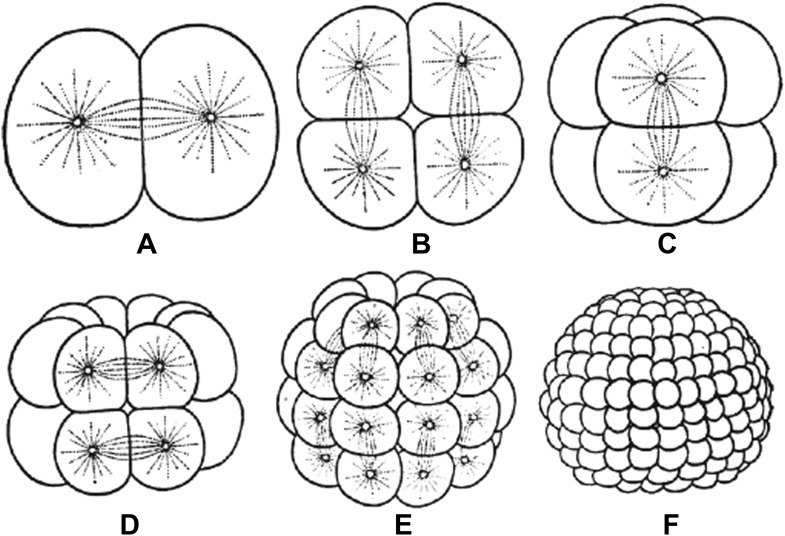
Embryos undergo multiple rounds of rapid division during the early stages of development of many animals. As the cells become progressively smaller, the spindles inside them also decrease in size. Reproduced from Wilson (1897).

FIGURE CREDIT: REPRODUCED FROM WILSON EB (1897).

To appreciate how fiendishly difficult this problem is, it is important to realize that the standard usage of two of the mainstays of modern cell biology research, namely inferring causation by 1) detecting co-occurrence of a protein and a phenotype and 2) perturbing the activity of a protein and examining the effect on a phenotype—cannot be used to rigorously establish the mechanisms controlling differences in spindle size. An extreme example illustrates this point: spindles are primarily composed of microtubules, which are in turn composed of the protein tubulin. Thus larger spindles contain more tubulin (co-occurrence) and depleting tubulin will reduce spindle size (perturbation), but this does not mean that changes in tubulin are responsible for changes in spindle size during development. Rather, these results suggest only that changes in tubulin *could* affect spindle size, not that they actually do so.

Wilbur and Heald overcome these difficulties through a powerful and conceptually straightforward approach: they prepare extracts from embryos at different developmental stages and assemble spindles in these extracts. They find that spindles in the extracts are the same size as the spindles in the embryos the extracts were made from, even though the extracts lack cell boundaries. This proves that changes in the size of the spindle are caused by changes in the state of the cytoplasm and are not directly controlled by cell size (at least in this system). This leads us naturally to the next question: how do changes in the cytoplasm produce changes in spindle size? To address this issue, Wilbur and Heald first characterize the behaviors of microtubules grown off of centrosomes—structures that nucleate microtubules—in the extracts. They observe that microtubule polymerization is similar in the different extracts, but that microtubules switch to a depolymerizing state, or ‘catastrophy’, at a higher rate in later stage extracts. It has been argued that modifying microtubule catastrophy rates can change spindle length (Ohi et al., 2007; Loughlin et al., 2010), presumably by altering the lengths of microtubules, suggesting that the decrease in spindle length during early development might be caused by the increase in catastrophy rate. However, differences in microtubule lengths cannot be the whole story because spindles from early stage extracts are not just longer; they are also wider and appear denser, suggesting that they contain far more microtubules than late stage spindles.

Next, Wilbur and Heald use a candidate approach to attempt to discover which cytoplasmic factors are responsible for the differing rates of microtubule catastrophies in the different extracts. They identify one protein known to increase microtubule catastrophies, namely the kinesin-13, kif2a, as being enriched on spindles in late stage extracts, and use perturbation experiments to argue that kif2a contributes to the differences in spindle size. However, the concentration of kif2a is the same in early and late stage extracts, which means that if kif2a is causing differences in the extracts, this must be because its activity is being regulated differently. Wilbur and Heald provide evidence that this regulation could be performed by importin α, which inhibits kif2a. They argue that over successive cell divisions importin α becomes increasingly sequestered in membranes, causing the cytoplasmic concentration of free importin α to decrease. This leads to an increase in kif2a activity, and thus an increase in microtubule catastrophy rates. This is a clever suggestion, as it offers a possible mechanism by which cell biochemistry could indirectly readout changes in cell size, since smaller cells have a greater surface to volume ratio than larger cells. However, it is not clear why importin α would bind to membranes only after they have been deposited to form cell boundaries, and not earlier when they are in cytoplasmic stores.

Demonstrating that the changes in spindle size during early development are driven by the changing biochemistry of the cytoplasm is a landmark finding that pushes the field forward and allows new, more precise questions to be formulated. One issue that will be important to resolve is the extent to which these changes are multifactorial. Are the differences in spindle structure primarily caused by one or two keys factors, or are large numbers of factors involved, each contributing a little, perhaps in opposing directions? Wilbur and Heald's study provides a hint that the situation may be complicated: they found that spindles from early stage extracts are enriched for the kinesin-13, MCAK. This suggests that MCAK may be more active in early developmental stages—a change that goes the ‘wrong’ way, as MCAK is known to increase catastrophy rates, whereas microtubules in the early stage extracts have a decreased rate of catastrophies***.*** In any case, more work remains in the young and challenging area of studying how cell biology differs in different cells.
